# Obesity paradox in subarachnoid hemorrhage: a systematic review

**DOI:** 10.1007/s10143-019-01182-5

**Published:** 2019-10-29

**Authors:** Ilari Rautalin, Jaakko Kaprio, Miikka Korja

**Affiliations:** 1 Department of Neurosurgery, University of Helsinki and Helsinki University Hospital, P.O. Box 266, FI-00029 Helsinki, Finland; 2 Department of Public Health, University of Helsinki, P.O. Box 20, FI-00014 Helsinki, Finland; 3 Institute for Molecular Medicine FIMM, P.O. Box 20, FI-00014 Helsinki, Finland

**Keywords:** Obesity, Mortality, Subarachnoid hemorrhage, Systematic review

## Abstract

As the number of obese people is globally increasing, reports about the putative protective effect of obesity in life-threatening diseases, such as subarachnoid hemorrhage (SAH), are gaining more interest. This theory—the obesity paradox—is challenging to study, and the impact of obesity has remained unclear in survival of several critical illnesses, including SAH. Thus, we performed a systematic review to clarify the relation of obesity and SAH mortality. Our study protocol included systematic literature search in PubMed, Scopus, and Cochrane library databases, whereas risk-of-bias estimation and quality of each selected study were evaluated by the Critical Appraisal Skills Program and Cochrane Collaboration guidelines. A directional power analysis was performed to estimate sufficient sample size for significant results. From 176 reviewed studies, six fulfilled our eligibility criteria for qualitative analysis. One study found paradoxical effect (odds ratio, OR = 0.83 (0.74–0.92)) between morbid obesity (body mass index (BMI) > 40) and in-hospital SAH mortality, and another study found the effect between continuously increasing BMI and both short-term (OR = 0.90 (0.82–0.99)) and long-term SAH mortalities (OR = 0.92 (0.85–0.98)). However, according to our quality assessment, methodological shortcomings expose all reviewed studies to a high-risk-of-bias. Even though two studies suggest that obesity may protect SAH patients from death in the acute phase, all reviewed studies suffered from methodological shortcomings that have been typical in the research field of obesity paradox. Therefore, no definite conclusions could be drawn.

**Electronic supplementary material:**

The online version of this article (10.1007/s10143-019-01182-5) contains supplementary material, which is available to authorized users.

## Introduction

Subarachnoid hemorrhage (SAH) is characterized as an acute cerebrovascular disease with multiple systemic complications and long intensive care unit (ICU) period [43]. Since roughly one-fourth [24] of SAH patients die suddenly before reaching a hospital ward and another one-fourth within the first 30 days [25], factors affecting the acute phase mortality are of interest.

Obesity is a well-known risk factor for increased morbidity and mortality in the general population [13, 14]. As the number of obese persons is globally increasing [10], a theory called the obesity paradox has aroused increasing interest in recent years. According to this theory, overweight and obese people may have a survival benefit in various acute illnesses and medical conditions. In stroke subtypes other than SAH, such as intracerebral hemorrhage (ICH) [7, 21] and ischemic stroke [20, 22, 35, 45, 47], being overweight or obese is associated with a favorable outcome. However, the true impact of BMI on the survival of any ICU patient group has remained unclear, and several studies have reported controversial results about the effects of obesity on the outcome of critically ill patients [1, 16, 31]. Since contributing comorbidities and a critical illness itself are presumably strong predictors of outcome, one of the major challenges in many previous obesity paradox studies has been the heterogeneity in the etiology of critical illnesses leading to ICU admission. Since only a few critical but relatively common acute illnesses among working-age and relatively healthy people invariably lead to long and expensive ICU periods that are further burdened with systemic complications, determining the role of obesity in survival has thus remained overly challenging.

SAH, which affects working-age and relatively healthy people, is an optimal acute and critical illness to study the effect of obesity on short-term mortality. Contrary to other stroke subtypes, namely ICH and ischemic stroke [29], neither systematic reviews nor meta-analyses have been conducted on the topic of SAH survival and obesity. Therefore, our purpose was to review the previous literature on the possible effect of obesity on SAH survival. Because some studies have suggested that the obesity paradox can be explained by selection bias, confounding factors and inappropriate study designs [4, 9, 12, 19, 41, 42], we also tried to evaluate the quality of previous studies. Our hypothesis was that the obesity paradox is true (i.e., obesity (metabolic reservoir capacity) protects critically ill patients from death), and that this protective effect can be investigated by studying SAH patients, who often have lengthy and potentially catabolic ICU stays.

## Materials and methods

Study protocol is available in the international prospective register of systematic reviews (PROSPERO, code CRD42018100003) and follows the checklist of Preferred Reporting Items for Systematic review and Meta-Analyses (PRISMA) [39] (Online Resource 1).

### Search strategy

Details of the literature search strategy are described in Online Resource 2. Briefly, the search was based on both keywords and index terms and utilized three different databases: PubMed, Scopus, and Cochrane library. A study question and eligibility criteria were formulated using the four-step PICO (Patients, Intervention, Controls, Outcome) principle [17]. Case reports, case series, letters, commentaries, book chapters, animal studies, and descriptive studies without calculated risk estimates (hazard ratio (HR), odds ratio (OR) or relative risk (RR)) were excluded.

### Quality of studies

To evaluate the heterogeneity and research methods of previous studies, we chose to use the Cochrane Collaboration Handbook and Critical Appraisal Skills Program (CASP) [15]. Based on the appropriate checklists, we focused on selection, detection, and measurement bias; limited and residual confounding; as well as on statistical power and causality between exposures and outcomes. To specify the sources of bias, we created six typical domains to describe these limitations wherein each domain had specific requirements classifying the studies into low, unclear or high-risk-of-bias categories (Online Resource 2). Finally, reviewed studies were grouped into either low-quality or high-quality categories. To reach the high-quality category, all six domains had to be categorized as low-risk-of-bias.

### Statistical analyses

A directional power analysis was performed to estimate sufficient sample size for significant results. The protective effects of being overweight, obese, and morbidly obese were optimistically estimated using an OR of 0.8 for BMI 25–29.9, OR = 0.7 for BMI 30–39.9, and OR = 0.6 for BMI ≥ 40, with a reference 30-day mortality figure for the normal weight category (BMI = 18.5–24.9) approximated to 25% for hospital-based studies and 40% for studies that also included sudden-death SAHs before hospitalization [25]. Standard values were used for significance (*p* < 0.05) and statistical power (*p* = 0.8). Additionally, depending on the amount of high-quality studies, *I*^2^ statistics for heterogeneity and random effects models for calculation of pooled estimates were prepared for possible meta-analysis.

## Results

**Fig. 1:**
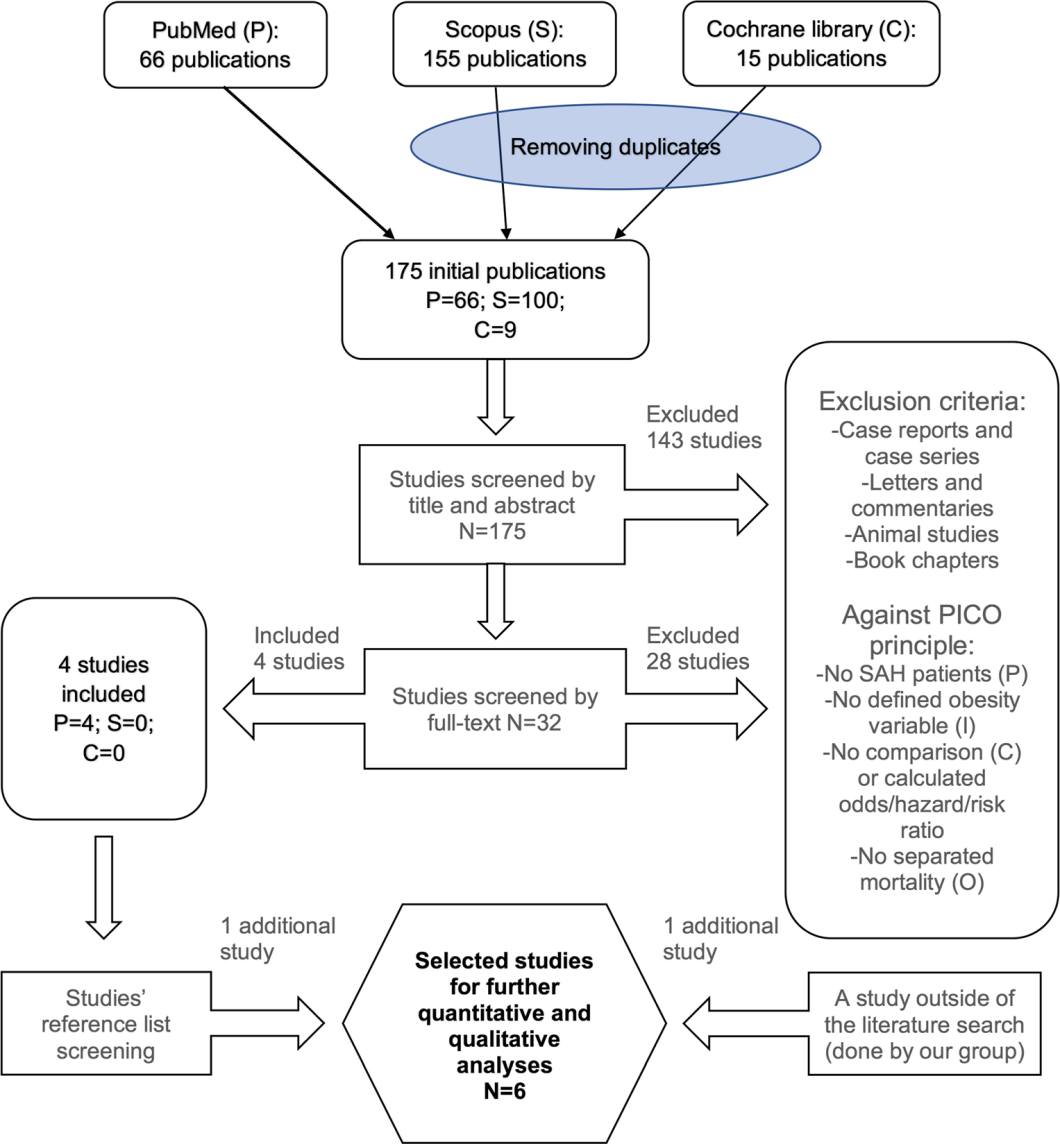
Flow chart of the literature search

The literature search is depicted in Fig. 1. The search yielded 175 articles, and 1 article [36] was found through reference list screening of the 175 articles. Of the 176 publications, 5 [8, 11, 18, 36, 46] fulfilled the eligibility criteria. Additionally, one study [27] outside of our literature search fulfilled the criteria and thus was added to further qualitative analysis. All 177 articles were published in English.

Study characteristics varied considerably (Table 1). Three studies [27, 36, 46] recruited cohorts and measured BMI values years before SAHs, whereas three [8, 11, 18] were based on hospital admission registers with BMI values measured upon admission.

**Table 1:** Study characteristics

First author	Country	Year	SAH cases	Mean age	Sex (% male)	Follow-up time	Obesity categories
Cohort studies with preictal follow-up							
Sandvei	Norway	2011	214	47.1	58.6	Before SAH: 16 years* After SAH: 6 months	BMI < 18.5 BMI 18.5–24.9 BMI 25–29.9 BMI ≥ 30
Yamada	Japan	2003	244	63.1	36.1	Before SAH: 9.9 years* After SAH: NR	BMI < 18.5 BMI 18.5–24.9 BMI ≥ 25
Lindbohm	Finland	2017	543	61.4	46.2	Before SAH: 23 years* After SAH: Before hospitalization	Continuous BMI
Cohort studies from hospital-based registers							
Dasenbrock	USA	2017	18,281	54.2	31.3	In hospital	NO (BMI < 30) Obese (BMI = 30–40) MO (BMI > 40)
Elliot	USA	2017	224,561	59.4	40.2	In hospital	NMO (BMI ≤ 40) MO (BMI > 40)
Hughes	USA	2015	305	55.8	35.2	Short-term (in hospital or 30 days after discharge) Long-term (24 months)	BMI < 25 BMI 25–30 BMI ≥ 30

*NR* not reported, *MO* morbidly obese patients, *NMO* nonmorbidly obese patients, *NO* nonobese patients

*Mean follow-up time

### Study 1

In a Norwegian study, Sandvei et al. [36] prospectively examined incidence, case fatality, and preictal risk factors for survival among 214 (59% male; mean age 47 years) SAH patients, including also sudden-death SAHs before hospitalization. Mean pre-SAH follow-up was 16 years, with post-SAH follow-up divided into short-term (3 and 30 days) and long-term (6 months). BMI was measured years before SAH at enrollment and categorized according to WHO guidelines. No re-measurements of BMI were performed at admission.

### Study 2

Yamada et al. [46] studied risk factors for fatal SAHs in a Japanese population-based prospective study. BMI calculations were based on self-administered questionnaires at enrollment, and BMI values were divided into low (BMI < 18.5), medium (BMI 18.5–24.9), and high (BMI ≥ 25) categories. During the follow-up of 1,086,963 person-years and a median follow-up time of 9.9 years, 244 (36% male; mean age 63 years) SAH deaths were reported. Mortality was verified from the national cause of death register. No post-SAH follow-up was performed, and no re-measurements of BMI were performed on admission.

### Study 3

In the third pre-SAH follow-up study, Lindbohm et al. [27] investigated risk factor differences between sudden-death and hospitalized SAH patients in a large prospective cohort study from Finland. During the follow-up of 1.52 million person-years and a median follow-up time of 23 years, 98 of 543 SAH patients (46% male; mean age 61 years) died outside of hospital wards. BMI was measured years before SAH and analyzed only as a continuous variable. Neither hospital follow-up nor re-measurement of BMI was performed.

### Study 4

Using the hospital-based register of the Nationwide Inpatient Sample (NIS) in the USA, Dasenbrock et al. [8] evaluated the association of both obesity (BMI 30–40) and morbid obesity (BMI > 40) with various outcomes after SAH. BMI categories were indirectly estimated by using registered ICD-9-CM diagnosis codes of obesity and morbid obesity, whereas controls consisted of NIS patients without either diagnosis. Between 2002 and 2011, 18,281 (31% male; mean age 54 years) SAH patients were included in the analyses of in-hospital mortality.

### Study 5

In another study based on the NIS, Elliott et al. [11] determined the impact of morbid obesity (BMI ≥ 40) on non-traumatic SAH outcomes. In-hospital mortality analyses were based on 224,561 (40% male; mean age 59 years) SAH patients diagnosed between years 2008 and 2013. BMI categories were also indirectly estimated by using registered ICD-9-CM codes of morbid obesity. NIS patients with BMI < 40 values served as a control group.

### Study 6

In the third study from the USA, Hughes et al. [18] examined the association of obesity with both short-term (in-hospital or within 30 days after hospital discharge) and long-term (> 24 months) outcomes. BMI was measured at hospital admission and analyzed as a continuous variable in univariate and multivariate analyses. The hospital-based study included 305 (35% male; mean age 56 years) SAH patients admitted between 2002 and 2011.

### Risk-of-bias and quality of studies

Based on the Cochrane Collaborator Handbook and CASP checklist, the selected domains for risk-of-bias estimation were the following: inclusion of sudden-death SAHs, valid obesity measurements, proper obesity analysis, control for confounding, comprehensive short-term follow-up, and sufficient sample size (Online Resource 2). None of the studies fulfilled the criteria for low risk-of-bias classification in all six domains; thus, all six studies [8, 11, 18, 27, 36, 46] were classified in the low-quality category (Table 2).

**Table 2:** Risk of bias evaluation. Plus sign (+) represents low-risk, NA (not applicable) unknown risk, and minus sign (−) high-risk-of-bias

First author	Sudden-death SAHs	Obesity measurement	Obesity analysis	Short-term follow-up	Confounding control	Sufficient sample size
Low-quality studies						
Sandvei	+	−	+	+	−	−
Yamada	NA	−	−	NA	+	−
Lindbohm	+	−	−	−	+	−
Dasenbrock	−	−	−	+	−	+
Elliot	−	−	−	+	−	+
Hughes	−	+	−	+	−	−

### Power analysis

Based on the pre-defined effect estimations and power analysis, we calculated that in studies with sudden-death SAHs (reference mortality 40%), the minimum cohort size for significant results was 1128 SAH patients (564 per category) when comparing overweight (BMI 25–30) and normal weight (BMI 18.5–24.9) patients (estimated OR = 0.8), 488 (244 per category) when comparing obese (BMI > 30) and normal weight patients (estimated OR = 0.7) and 266 (133 per category) when comparing morbidly obese (BMI > 40) and normal weight patients (estimated OR = 0.6). Additionally, in hospital-based studies (reference mortality 25%), the minimum cohort sizes were 1094, 466, and 250, respectively. Based on these criteria, only two [8, 11] of the six studies consisted of an adequate sample size to reach sufficient statistical power (Table 2).

### Short-term mortality

The definition of “short term” varied between studies (Table 1). Only the Norwegian study [36] included sudden-death SAHs before hospitalization in their short-term (30-day) follow-up. However, contrary to Lindbohm et al. [27], no separate association analyses for outside hospital deaths were performed. Three [8, 11, 18] studies, all conducted in the USA, started the follow-up from hospital admission. All these studies assessed in-hospital mortality rates and associations, but one study also included deaths until 30 days post-discharge.

Two studies [11, 18] found an association between BMI values and short-term SAH mortality (Table 3). First, Elliot et al. [11] found an inverse association (obesity paradox) between morbid obesity (BMI > 40) and SAH-related in-hospital mortality (OR=0.83) in a multivariate model (Table 3). Second, Hughes et al. [18], with a somewhat longer follow-up of up to 30 days post-discharge, also found an inverse association (obesity paradox) between SAH mortality and increasing BMI values (Table 3) in both univariate (OR = 0.91) and multivariate (OR = 0.90) models. The study reached statistical significance despite our power analysis suggested that the cohort size was inadequate to reach significance. Two other studies [8, 36] concluded that no significant associations existed between BMI and short-term SAH mortality. The last two studies [27, 46] did not define the time frame of short-term follow-up.

**Table 3:** SAH studies reporting associations between BMI and mortality

First author	Follow-up	Analyses by sex	BMI categories (kg/m^2^)	Risk of mortality HR/OR (95% CI)	
				Univariate analysis	Multivariate analysis
Short-term mortality					
Sandvei	3 days	–	Underweight (< 18.5)	–	–
			Normal weight (18.5–24.9)	–	Reference
			Overweight (25–29.9)	–	0.6 (0.3–1.4)
			Obese (≥ 30)	–	1.1 (0.4–3.1)
	30 days	–	Underweight (< 18.5)	–	–
			Normal weight (18.5–24.9)	–	Reference
			Overweight (25–29.9)	–	1.1 (0.6–2.0)
			Obese (≥ 30)	–	0.9 (0.3–2.1)
Dasenbrock	In hospital	–	Non–obese (< 30)	Reference	Reference
			Obese (30–40)	0.84 (0.65–1.09)	0.90 (0.69–1.18)
			Morbidly obese (> 40)	0.75 (0.54–1.06)	0.77 (0.54–1.11)
Elliot	In hospital	–	Nonmorbidly obese (≤ 40)	–	Reference
			Morbidly obese (> 40)	–	0.83 (0.74–0.92)
Hughes	In hospital or 30 days after first discharge	–	Continuously	0.91 (0.84–0.98)	0.90 (0.82–0.99)
Long-term mortality					
Sandvei	6 months		Underweight (< 18.5)	–	1.6 (0.1–28.4)
			Normal weight (18.5–24.9)	–	Reference
			Overweight (25–29.9)	–	1.0 (0.5–1.9)
			Obese (≥ 30)	–	1.0 (0.4–2.4)
Hughes	> 24 months	–	Continuously	0.92 (0.86–0.97)	0.92 (0.85–0.98)
No reported follow-up					
Yamada	NR	Both	Low (< 18.5)	1.63 (1.06–2.50)	1.82 (0.98–3.38)
			Moderate (≥ 18.5 and < 25.0)	Reference	Reference
			High (≥ 25.0)	1.02 (0.73–1.43)	–
		Men	Low (< 18.5)	1.85 (0.91–3.75)	2.72 (1.03–7.23)
			Moderate (≥ 18.5 and < 25.0)	Reference	Reference
			High (≥ 25.0)	0.73 (0.38–1.38)	–
		Women	Low (< 18.5)	1.52 (0.89–2.60)	1.44 (0.64–3.21)
			Moderate (≥ 18.5 and < 25.0)	Reference	Reference
			High (≥ 25.0)	1.16 (0.79–1.73)	–
Lindbohm	Before hospitalization	–	Continuously	–	0.86 (0.68–1.09)

*BMI* body mass index, *CI* confidence interval, *HR* hazard ratio, NR not reported, *OR* odds ratio

### Long-term mortality

In addition to short-term mortality, two studies also analyzed long-term mortality (Table 3). Only Hughes et al. [18] found an inverse association (obesity paradox) between continuously increasing BMI values and long-term (> 24 months) post-SAH mortality in both univariate (OR = 0.92) and multivariate (OR = 0.92) models (Table 3).

### Mortality without a defined time frame of follow-up

Two studies [27, 46] did not define a follow-up time for hospitalized patients. First, Yamada et al. [46] found an association between underweight (BMI < 18.5) patients and fatal SAHs in general (Table 3). In a univariate analysis, the association was reported for both sexes (HR = 1.63), whereas in the multivariate analysis, low BMI remained an independent risk factor only for men (HR = 2.73) (Table 3). The study found no significant associations between overweight/obesity (BMI ≥ 25) and mortality when using normal weight patients as a reference group (Table 3). Second, Lindbohm et al. [27] reported an inverse association between increasing BMI and all SAH cases (HR = 0.90). However, when analyses were done separately for suddenly died and hospitalized SAH patients, no significance was reached. Additionally, in comparison analysis, no BMI difference was found between sudden-death and hospitalized SAH patients (*p* = 0.81 for men, *p* = 0.76 for women).

### Meta-analysis

Due to methodological differences and the absence of high-quality studies, a quantitative meta-analysis was impractical to conduct.

## Discussion

Two studies [11, 18] found an obesity paradox in SAH mortality, but methodological shortcomings relegate these studies into a high-risk-of-bias category; therefore, firm conclusions about a protective effect of obesity in SAH mortality cannot be drawn. Although the reported obesity paradox can be explained at least partly by methodological shortcomings, physiological mechanisms explaining better survival of obese patients are also possible. For example, protective mechanisms might relate to an increased metabolic reserve of adipose or muscle mass, which could improve tolerance of major catabolic and inflammatory events among critically ill patients treated in ICUs. Supporting this theory, a few studies [3, 8, 34] have related obesity to more favorable outcomes, measured using the modified Rankin Scale (mRS), and milder overweight to lower complication rates as well. On the other hand, other studies have reported contradictory findings [33, 37, 44].

While a protective effect of obesity in SAH survival is still questionable, morbidly obese SAH patients in particular seem to have several disadvantages. In SAH, postoperative infections [8], venous thromboembolisms [8, 26, 38], acute respiratory failure [11], fever [32], prolonged hospitalization [11], and non-routine discharge [8] are associated with higher BMI. Since obese patients tend to have higher complication rates and longer hospital stays, studies on SAH outcome may have a performance bias relating to differing short-term treatments. In addition to higher complication rates, morbid obesity increases hospital costs in SAH [11]. However, if the obesity paradox is true, the higher treatment costs and longer hospitalizations of obese or morbidly obese patients could be better justified.

In other stroke subtypes, sudden deaths before hospitalization are less common than in SAH [5, 40]. In SAH, nearly half of 1-year deaths occur before hospitalization and around 90% within the first 30 days [25]. Since hospitalized SAH patients often stay 2–3 weeks in the ICU and the majority of deaths occur within the first month following the ictus, a follow-up time of 30 days seems optimal in assessing any effects of obesity on mortality in SAH. Moreover, since less than 5% of SAH patients die between 30 days and 1 year [25], SAH appears to be a rather optimal acute disease to examine any effects of obesity on the short-term survival of critically ill patients.

### Limitations in previous studies

Four studies [8, 11, 18, 46] excluded sudden-death SAHs. Studies missing roughly 25% of all SAHs and about 50% of fatal cases have a theoretical risk for selection bias. Even though one previous study did not find BMI differences between sudden-death and hospitalized SAH patients [27], no comparison was made between fatal cases only. Since the same study found that sudden-death SAH patients have significantly worse overall risk factor profiles than the hospitalized patients [27], even a minimal BMI difference between sudden SAH deaths and hospitalized fatal SAHs may distort hospital-based registers to the extent of losing study reliability. In the Norwegian study with both sudden and hospitalized SAH deaths in the analyses [36], no comparisons between the groups were made.

With regard to obesity assessments, all six studies had several shortcomings. First, three studies [27, 36, 46] measured BMI only at enrollment. Since most adults gain weight until serious illnesses late in life or until the onset of sarcopenia [28], those who were overweight at enrollment are likely to also be overweight or obese at the onset of SAH. Therefore, the use of premorbid BMI is likely underestimating the effect of obesity. To avoid false negative results, future studies with long preictal monitoring periods could perform BMI remeasurements at admission. Second, two studies analyzed obesity as a continuous variable [18, 27], while three used only combined BMI categories, such as non-obese (BMI < 30) [8], non-morbidly obese (BMI < 40) [11], or high BMI (BMI > 25) [46] categories. Therefore, statistical analyses of weight extremes could not be conducted. Since the effect of obesity is unlikely linear (i.e., the fatter the better) and differences by obese severity have also been reported in SAH mortality, the comparison between overweight (BMI 25–29.9), obese (BMI 30–39.9), and morbidly obese (BMI ≥ 40) SAH patients is difficult to conduct if, for example, all patients with BMI values over 25 are combined into one category. Third, two studies estimated BMIs by relying on physician reports [8, 11], and one study used self-administered questionnaires [46] without controlling for measurement bias. All studies used only BMI as an obesity variable. Since BMI sensitivity has been reported to be only 50% for high mass of body fat [30], future studies could also consider other obesity variables with better sensitivity such as waist and hip circumference measurements, magnetic resonance imaging (MRI), dual-energy X-ray absorptiometry (DEXA), or bio-impedance of body.

Only two studies, NIS register-based studies [8, 11] from the USA, fulfilled the criteria of sufficient sample size providing high statistical power for analyses. Any protective effect of high BMI values is likely smaller than our optimistic estimation, so the other four studies [18, 27, 36, 46] likely had insufficient power to achieve significant results. In addition, our directional analysis did not consider effects of potential confounding, thus further underestimating the required cohort sizes.

### Confounders in studies on the obesity paradox in SAH

In a recent large prospective follow-up study, Lindbohm et al. [27] found that the risk for sudden SAH death seems to be highest among people with the worst risk factor profile (i.e., among heavy smokers and people with high blood pressure values). Both smoking [6] and high blood pressure values [14] have been associated with obesity. In terms of smoking, a large study of nearly 300,000 participants [19] reported that the observed obesity paradox in patients with cardiovascular diseases vanished after analyzing only non-smokers. For these reasons, we presumed that both smoking and blood pressure should be treated as confounders in low-risk-of-bias studies of the obesity paradox in SAH. Overall, two studies [27, 46] fulfilled this criterion (Tables 2 and 3).

As potential other confounders, obese patients often suffer from metabolic syndrome, diabetes, coronary artery disease, and peripheral vascular disease. Although these comorbidities might also impact the survival of critically ill patients, no high-quality evidence exists, suggesting an association between these comorbidities and short-term SAH mortality. Moreover, as the prevalence of these comorbidities is low in SAH patients [2, 23], we did not consider these factors as likely confounders, and therefore, they were not regarded mandatory in statistical analyses to reach the classification of low-risk-of-bias. In contrast, other factors, such as SAH severity and rebleeding, have been associated with higher mortality rates; no evidence relates these findings to obesity groups. Furthermore, Rinaldo et al. [34] found an effect of BMI on SAH outcome, and this effect varied depending on the treatment modality (clipping versus coiling). However, the study found no differences after using the validated WHO categories for BMI. In addition, the characteristics between treatment groups varied significantly. Since convincing evidence of treatment confounding in SAH does not exist, we did not include this factor in bias classifications. In brief, future studies about obesity paradox in SAH should try to consider at least smoking and hypertension as potential confounders and perhaps evaluate if composite variables of cardiovascular risk modify the relation of BMI and SAH mortality.

Although the literature search was done systematically utilizing three different databases, we may have missed some relevant publications. For example, large studies with comprehensive and wide-ranging risk factor assessments may have neglected minor and especially negative findings in their indexing. This type of reporting and indexing bias may cause the exclusion of studies like the one by Lindbohm et al. [27]. Furthermore, we used only English search terms, assuming that clinically relevant studies have been conducted in large hospitals and subsequently reported in English. Lastly, we were not able to conduct a meta-analysis due to methodological differences and the absence of high-quality studies.

## Conclusions

None of the reviewed obesity paradox studies were confirmative, and effects of obesity on the risk of death among critically ill SAH patients remain to be studied in more detail. However, since the poor-quality evidence suggests that the obesity paradox may exist in SAH and since the number of obese patients is globally increasing, future studies on the topic are encouraged. To avoid major biases, future studies should perhaps try to include sudden-death SAHs in their analyses, use valid and timely obesity measurements on all SAH patients, and aim for reasonable cohort sizes.

## Electronic supplementary material

Online Resource 1: PRISMA checklist (PDF 266 kb)

Online Resource 2: Supplemental Methods. Literature search, search strategy and quality assessment. (PDF 197 kb)
