# Supplementary material for: Obesity paradox in subarachnoid hemorrhage: a systematic review
Source: Neurosurg Rev. 2019 Oct 29;43(6):1555–63. doi: 10.1007/s10143-019-01182-5 (PMC7680302; doi:10.1007/s10143-019-01182-5)
Supplement: Supplementary file 2 — Supplemental Methods. Literature search, search strategy and quality assessment. (PDF 197 kb) [file 10143_2019_1182_MOESM2_ESM.pdf]

Obesity paradox in subarachnoid hemorrhage: a systematic review

Ilari Rautalin, BM[1,2]; Jaakko Kaprio, MD, PhD[2,3]; Miikka Korja, MD, PhD[1]

[1]Department of Neurosurgery, University of Helsinki and Helsinki University Hospital, P.O. Box 266, FI-00029 Helsinki, Finland

[2]Department of Public Health, University of Helsinki, P.O. Box 41, FI-00014 Helsinki, Finland

[3]Institute for Molecular Medicine FIMM, P.O. Box 20, FI-00014 Helsinki, Finland

Correspondence to Ilari Rautalin, Department of Neurosurgery, University of Helsinki, P.O. Box 266, FI-00029 Helsinki, Finland; E-mail address: ilari.rautalin@helsinki.fi; Telephone: +358 947187604 Fax: +358 947187616; ORCID: 0000-0002-6283-0398

Journal: Neurosurgical Review

Supplementary Material / Online Resource 2

Literature search

PubMed

Preliminary search 20th of February in 2018

(subarachnoid hemorrhage) AND (body mass index OR obesity) AND (mortality)

Publications: 33

Final search 1st of March in 2018 (updated 5th of December 2018)

(subarachnoid hemorrhage) AND (body mass index OR obesity OR BMI OR overweight) AND (mortality OR outcome)

Publications: 63 (66 in update)

Scopus

Final search 1st of March in 2018 (updated 5th of December 2018)

TITLE-ABS-KEY ("subarachnoid hemorrhage" AND (obesity OR "body mass index" OR bmi OR overweight) AND (mortality OR outcome))

Publications: 146 (155 in update)

Cochrane Library

Final search 1st of March in 2018 (updated 5th of December 2018)

(subarachnoid hemorrhage) AND (obesity OR body mass index OR BMI OR overweight) [Title, Abstract, Keywords]

Publications: 15 (15 in update)

Search strategy

We defined a specific study question in the PICO format (Population, Intervention, Comparison and Outcome). The following question was formulated: Do obese individuals have a lower risk than normal weight individuals of dying from subarachnoid hemorrhage? Based on this question, we formed a search strategy utilizing three different databases: PubMed, Scopus and Cochrane library.

Our search strategy included a preliminary search in the PubMed database for estimating the number of potential studies. Furthermore, primary search results were used to define proper index and keywords for final searches in all three databases. In final searches, we used both keywords and index terms. The preliminary search was done on the 20th of February 2018 and final searches on the 1st of March in 2018. Final searches were updated on the 5th of December 2018. Searches were done by one author and discussed together with second and third authors. An information specialist was consulted for an objective opinion of search strategy and for forming a proper study question.

The PICO format was also used to determine inclusion criteria: aneurysmal, non-traumatic SAH patients (P), reported obesity variable (I) and reference group for comparison (C) were all mandatory for inclusion. Additionally, mortality (O) was defined as an outcome measure. Case reports, case series, letters, commentaries, book chapters and animal studies were excluded. Descriptive studies without calculated odds ratios (ORs), hazard ratios (HRs) or risk ratios (RRs) were also excluded. English was the language used in literature searches.

Quality of selected studies

The quality of selected studies was evaluated systematically by the Cochrane Collaborator Handbook[1] and Critical Appraisal Skills Program (CASP)[2]. Based on the checklists, we created six specific domains in which study biases and methodological shortcomings were most likely and influential. Depending on whether the study fulfilled the criteria, studies were classified into low, unclear or high-risk-of-bias by each domain. To reach the overall classification of high-quality, all six domains had to fulfill low-risk-of-bias criteria.

1. Sudden-death SAHs

Since half of fatal SAHs occur before hospitalization, there is a risk for selection bias in studies using hospital-based registers. Since the risk factor status seems to be the worst among SAH patients dying before hospitalization, inclusion of sudden-death SAHs was required to reach low-risk-of-bias in this domain.

2. Obesity measurement

The second domain evaluated the potential risk for measurement bias. To reach the classification of low-risk-of-bias, obesity had to be measured by validated and generally known variables, such as BMI, and the measurements had to be performed at the time of SAH (for example, at admission).

3. Obesity analysis

Since obesity and SAH-related mortality have a possibly nonlinear association, combining extreme weight categories into a normal weight category or analyzing obesity only as a continuous variable may also bias analyses. Thus, we created the domain to evaluate valid analysis for obesity variables. To reach the classification of low-risk-of-bias, different categories for underweight, normal weight, overweight and obese/morbidly obese (e.g., according to WHO guidelines) were required.

4. Comprehensive short-term follow-up

Since the majority of fatal SAHs seems to occur within the first month[3], we selected comprehensive short-term follow-up as a domain to avoid possible detection bias. Either in-hospital, 30-day or a comparable follow-up time was required to achieve the classification of low-risk-of-bias.

5. Confounding control

Because consideration of all general risk factors for short-term mortality is overly challenging, we decided to focus on factors that have been previously associated with both higher/sudden SAH mortality and obesity. Smoking and high blood pressure are reported by high-quality studies[4-6] to interfere with both factors; therefore, to reach the classification of low-risk-of-bias in the domain of confounding control, these two variables needed to be adjusted for in multivariate analysis. Even though several other variables, such as SAH severity, amount of bleeding, aneurysm location and size, intracerebral hemorrhage, rebleeding and multiple post-operative complications, have been related to higher mortality after SAH, we excluded them in our quality assessment due to a lack of evidence suggesting that these variables associate with obesity categories.

6. Sufficient sample size

Since the theoretical protective effect of obesity is unlikely very strong, we included sufficient sample size as a domain to evaluate possible biases. Calculations for significant results were based on directional power analysis. Details are described under the subtitle Statistical analysis.

Supplemental references

1. Higgins JPT GS. Cochrane Handbook for Systematic Reviews of Interventions Version 5.1.0 [updated March 2011] [online].
2. Critical Appraisal Skills Programme (CASP). CASP Checklists (http://media.wix.com/ugd/dded87_e37a4ab637fe46a0869f9f977dacf134.pdf) [online].
3. Korja M, Silventoinen K, Laatikainen T, Jousilahti P, Salomaa V, Kaprio J. Cause-specific mortality of 1-year survivors of subarachnoid hemorrhage. Neurology 2013;80:481-486.
4. Lindbohm JV, Kaprio J, Jousilahti P, Salomaa V, Korja M. Risk Factors of Sudden Death From Subarachnoid Hemorrhage. Stroke 2017;48:2399-2404.
5. Carreras-Torres R, Johansson M, Haycock PC, et al. Role of obesity in smoking behaviour: Mendelian randomisation study in UK Biobank. BMJ 2018;361:k1767.
6. Guh DP, Zhang W, Bansback N, Amarsi Z, Birmingham CL, Anis AH. The incidence of co-morbidities related to obesity and overweight: a systematic review and meta-analysis. BMC Public Health 2009;9:88.
